# The Use of Stable Zinc Isotope Soil Labeling to Assess the Contribution of Complex Organic Fertilizers to the Zinc Nutrition of Ryegrass

**DOI:** 10.3389/fpls.2021.730679

**Published:** 2021-12-21

**Authors:** Bo-Fang Yan, Thilo Dürr-Auster, Emmanuel Frossard, Matthias Wiggenhauser

**Affiliations:** Department of Environmental Systems Science, Group of Plant Nutrition, ETH Zurich, Lindau, Switzerland

**Keywords:** manure, sewage sludge, plant nutrition, source tracing, DGT (diffusive gradients in thin films)

## Abstract

Manure and sewage sludge are known to add significant amounts of zinc (Zn) and other metals to soils. However, there is a paucity of information on the fate of Zn that derives from complex organic fertilizers in soil–plant systems and the contribution of these fertilizers to the Zn nutrition of crops. To answer these questions, we grew Italian ryegrass in the presence of ZnSO_4_, sewage sludge, and cattle and poultry manure in an acidic soil from Heitenried, Switzerland, and an alkaline soil from Strickhof, Switzerland, where the isotopically exchangeable Zn had been labeled with ^67^Zn. This allowed us to calculate the fraction of Zn in the shoots that was derived from fertilizer, soil, and seed over 4 successive cuts. In addition, we measured the ^67^Zn:^66^Zn isotope ratio with the diffusive gradients in thin films technique (DGT) on soils labeled with ^67^Zn and incubated with the same fertilizers. After 48 days of growth, the largest fraction of Zn in the ryegrass shoots was derived from the soil (79–88%), followed by the Zn-containing fertilizer (11–20%); the least (<2.3%) came from the seed. Only a minor fraction of the Zn applied with the fertilizer was transferred to the shoots (4.7–12%), which indicates that most of the freshly added Zn remained in the soil after one crop cycle and may thereby contribute to a residual Zn pool in the soil. The ^67^Zn:^66^Zn isotope ratios in the DGT extracts and the shoots measured at cut 4 were identical, suggesting that the DGT and plant took up Zn from the same pool. The proportion of Zn derived from the fertilizers in the DGT extracts was also identical to that measured in ryegrass shoots at cut 4. In conclusion, this work shows that stable Zn isotope labeling of the soil available Zn can be used to precisely quantify the impact of complex organic fertilizers on the Zn nutrition of crops. It also demonstrates that DGT extractions on labeled soils could be used to estimate the contribution of Zn fertilizers to plant nutrition.

## Introduction

Complex organic fertilizers such as sewage sludge, animal manure, and compost are known sources of heavy metals such as zinc (Zn) in agroecosystems (Nicholson et al., 2003; Imseng et al., 2019). Bolan et al. (2004) reported that the concentration of Zn in animal manure and sewage sludge ranged from 1.8 to 6480 mg kg^−1^ (median 378 mg kg^−1^). Tella et al. (2016) and Hodomihou et al. (2020) reported that Zn was mainly present as inorganic species in organic fertilizers, including Zn adsorbed on iron oxides and amorphous Zn-phosphate. Some of these Zn species can contribute directly to the pool of soil Zn available and be taken up by crops. Complex organic fertilizers can also increase soil Zn availability through the inputs of carbon (C), nitrogen (N), or other elements and thereby raise Zn uptake by plants (Aghili et al., 2014). These authors demonstrated that the application of plant residues labeled with ^65^Zn strongly enhanced the uptake of Zn derived from the soil (non-labeled) by wheat. An increased solubility of soil Zn may be caused by nitrification of ammonium added with the organic fertilizer, which leads to acidification, and also by the decomposition of organic compounds leading to the release of water-soluble organic compounds that can form complexes with soil Zn (Gramlich et al., 2013; Soltani et al., 2014). Meanwhile, mineral nutrients and C inputs into soils may also promote plant uptake of Zn by favoring plant growth or improving soil structure, which allows better soil exploration by roots (Mäder et al., 2002; Alloway, 2009). To date, the effect of organic fertilizers on the plant uptake of soil Zn, and their contribution to Zn uptake by plants through Zn supply, has been quantified only for fresh plant residues (Aghili et al., 2014) but not yet for animal manure or sewage sludge.

The amount of Zn derived from a fertilizer that is taken up by a plant can be estimated by calculating its “apparent use efficiency” (AUE). AUE is the difference between the amount of Zn taken up by a plant that has been fertilized with Zn and the amount of Zn taken by the same plant grown in the absence of Zn fertilizer, divided by the amount of Zn added as fertilizer. Using this mass balance approach, Berenguer et al. (2008) estimated that 9–22% of the Zn added by liquid swine manure was taken up by maize plants in the year following the input. However, this approach rests on the implicit hypothesis that Zn derived from the soil is independent from the fertilizer addition (Schindler and Knighton, 1999). In other words, the AUE approach ignores the aforementioned effects of organic fertilizers on the solubility of Zn in the soil. To determine the fate of freshly applied Zn, therefore, the plant uptake of Zn derived from organic fertilizer and soil needs to be distinguished, which can be precisely done using Zn isotopes.

Quantifying the amount of Zn taken up by a plant from freshly applied fertilizer or soil can be achieved using isotopic approaches whereby either the fertilizer Zn or the soil available Zn is isotopically labeled. Whereas water soluble Zn or plant residues can be easily labeled homogeneously with radioactive or stable Zn isotopes, it is very difficult to do this with animal manure and sewage sludge, since they comprise mixtures of different inorganic and organic Zn phases (Bosshard et al., 2011; Hodomihou et al., 2020). Instead of labeling the fertilizer, it is possible to label the soil available Zn and calculate the amount of Zn derived from soil, fertilizer, and seed by analyzing the isotope ratio of Zn taken up by plants, for example, as done for phosphorus (P) by measuring the decrease of P-specific activity in plants (Pypers et al., 2006; Frossard et al., 2011). A recent methodological study has been shown that labeling soils with the stable isotope ^67^Zn can be used to assess the plant uptake of Zn derived from wheat straw compost (Dürr-Auster et al., 2019). This requires that the isotope ratios of ^67^Zn:^66^Zn can be adequately resolved which was achieved using a quadrupole single-collector (Q-)ICP-MS. Hence, labeling the pool of soil exchangeable Zn with ^67^Zn could be used to quantify the plant uptake of Zn derived from the soil and from sewage sludge or animal manure. Moreover, the isotope label can be used to calculate the recovery of the Zn applied with the fertilizer in the plant. Such recovery calculations can provide valuable insights about the potential fate of Zn in soil–plant systems after harvest (McBeath et al., 2013; Bracher et al., 2021; Mattiello et al., 2021).

The diffusive gradients in thin films technique (DGT) is a well-established tool to predict the plant available pool of Zn in the soil. A strong correlation between the amount of Zn extracted by the DGT sampler and the concentration of Zn in plant tissues has been shown in several studies (e.g., Degryse et al., 2009; Tandy et al., 2011). By measuring the isotopic composition of DGT extract in fertilized soils labeled with Zn isotope, it should be possible to identify the contribution of different sources (soil, fertilizer) in the available Zn pool independently from the effect of the plants (e.g., dry matter production, root exudates, seed Zn, etc.). However, this type of analysis has not yet been done for Zn.

The objectives of this study were to (1) precisely quantify the amount of Zn in plants derived from fertilizer, soil, and seeds in soils amended with distinct complex organic fertilizers, (2) determine the fate of Zn input with complex organic fertilizer in soil–plant systems, and (3) test whether DGT can be employed to predict the relative contribution of soil and fertilizer sources to the plant uptake of Zn. To this end, two cropped soils, an acidic soil from Heitenried, Switzerland, and an alkaline soil from Strickhof, Switzerland, were labeled with ^67^Zn. Italian ryegrass (*Lolium multiflorum*, var. Gemini) was grown as a model plant that takes up most of its Zn from the soil isotopically exchangeable pool (Sinaj et al., 2004). Besides a control that received no Zn, water-soluble ZnSO_4_, sewage sludge, poultry manure, and cattle manure were applied. These fertilizers brought similar amounts of Zn but different amounts of N and C to the soils.

## Materials and Methods

### Soils and Zinc Fertilization

The soils were collected, and the isotopically exchangeable Zn was labeled according to the procedure described in Dürr-Auster et al. (2019). Briefly, the arable layer of two soils (0–20 cm depth) was sampled from Heitenried and Strickhof, both in Switzerland. These two soils were selected based on their distinct pH, since this property is a major factor affecting the solubility of soil Zn and plant Zn uptake (Mertens and Smolders, 2013). Both soils were percolated with a nutrient solution containing ^67^Zn-enriched isotope (^67^Zn abundance = 32%) used in a wheat growth trial (Signorell et al., 2019) for 20 weeks. Then, they were mixed, sieved to 7 mm aggregate size, air-dried, and stored in dark and dry conditions at room temperature for 1 year before being used for the experiments presented here. The properties of the soil after the labeling procedure are summarized in Table 1.

**Table 1:** Selected soil properties after the labeling procedure.

**Origin^a^**	Soil type^b^^,^^*^	**Texture**	pH^*^ (H_**2**_O^c^)	Total Zn^*^ (EDXRF^d^)	DTPA^e^-extractable Zn^*^	Water-holding capacity^f^^,^^*^	**CaCO_**3**_**
				**mg Zn kg^−1^ DM^g^ soil**	**mg Zn kg^−1^ DM soil**	**g H_**2**_O kg^−1^ DM soil**	**g kg^−1^ DM soil**
Heitenried	Fluvisol	Sandy loam	4.9	54.1	4.1	387	0
Strickhof	Cambisol	Loam	7.7	101.1	5.2	447	36.7

* *Data were published in Dürr-Auster et al. (2019)*.

a *Collected from a depth of 0–20 cm*.

b *According to the International Union of Soil Sciences (IUSS) Working Group World Reference Base for Soil Resources (IUSS Working Group WRB, 2015)*.

c *pH in H_2_O with 1/2.5 (m/v) solid/liquid ratio*.

d *Energy dispersive X-ray fluorescence spectrometry*.

e *Diethylenetriaminepentaacetic acid*.

f *Maximal soil saturation with H_2_O without external pressure*.

g *Dry matter*.

The dried sewage sludge was obtained from the wastewater treatment facility of the city of Zurich, Switzerland (https://www.stadt-zuerich.ch/ted/de/index/entsorgung_recycling/sauberes_wasser/klaerwerk.html). Poultry manure and cattle manure in the form of dried pellets were bought from the Landi farmers' shop (https://www.landi.ch). These organic fertilizers were finely ground before their application. Mineral Zn fertilizer was supplied as a ZnSO_4_ solution. These Zn-containing fertilizers were thoroughly mixed with each labeled soil. The application rate was based on the Zn concentration of the fertilizer, leading to similar amounts of Zn added per kilogram of soil (i.e., Zn input) but different N and C inputs among these treatments (Table 2). A control treatment that received no Zn was also established.

**Table 2:** Total Zn concentration in the studied fertilizers and the input of total Zn, N, and C to soil with the application of each fertilizer.

**Treatment**	**Zn concentration in fertilizers^a^**	**Zn input with fertilization**	**N input with fertilization^b^**	**C input with fertilization^b^**
	**mg Zn kg^−1^ fertilizer**	**mg Zn kg^−1^ DM^c^ soil**	**mg N kg^−1^ DM soil**	**mg C kg^−1^ DM soil**
No Zn	0	0	0	0
ZnSO_4_^d^	440 × 10^3^	1.42	0	0
Sewage sludge^e^	634	1.52	90	0.734 × 10^3^
Poultry manure^e^	443	1.51	88	1.090 × 10^3^
Cattle manure^e^	353	1.56	225	1.704 × 10^3^

a *Measured in HNO_3_ microwave digested fertilizers (Dürr-Auster et al., 2019)*.

b *Determined through CN elemental analysis (Thermo Scientific Flash EA 1112)*.

c *Dry matter*.

d *Applied as water solution*.

e *Applied as dried powder*.

### Plant Growth Trial

The plant growing conditions were similar to those described in Dürr-Auster et al. (2019). Briefly, pots containing 400 g labeled dry soil with or without the addition of the abovementioned Zn-containing fertilizers were watered to 40% water-holding capacity and placed randomly in a growth chamber (14 h day–light at 25 klx, day–night temperature 24–18°C, and relative atmospheric humidity 60–65%). Then 500 mg of Italian ryegrass seeds (35.2 μg Zn g^−1^ seed) was sown in each pot. As observed in our previous isotope labeling studies on phosphorus and Zn, the root system of ryegrass can colonize well in a pot with a relatively small volume of soil over a short growth time, which is a prerequisite so that the plant shoots can provide a proper estimation of the isotopic signature of the available P and Zn (Sinaj et al., 2004; Frossard et al., 2014). Ten days after sowing, a nutrient solution was applied at a rate of 506 mg N kg^−1^ soil (ammonium nitrate), 55 mg P kg^−1^ soil (phosphate), 138 mg K kg^−1^ soil (K^+^), 31 mg S kg^−1^ soil (sulfate), 23 mg Mg kg^−1^ soil (Mg^2+^), 5 mg Fe kg^−1^ soil (Fe-EDTA), 232 μg B kg^−1^ soil (boric acid), 127 μg Mn kg^−1^ soil (Mn^2+^), 31 μg Cu kg^−1^ soil (Cu^2+^), and 54 μg Mo kg^−1^ soil (molybdic acid). Distilled water was supplied regularly to the pot by weighing to maintain soil moisture between 40 and 80% water-holding capacity. No drought symptoms were observed on the ryegrass plants throughout the experiment. Each treatment had 4 replicates with 1 pot per replicate. These pots were randomly rearranged after each watering. The shoots were harvested 4 times by cutting at 21, 30, 38, and 48 days after sowing, to minimize the influence of seed Zn on the tracing of fertilizer Zn. After each cut, the nutrient solution was resupplied at a rate of 20% of the first load.

### Incubation Experiment

A soil incubation experiment was carried out in parallel with the growth trial for deploying the DGT sampler to extract Zn from the soil available Zn pool. To this end, one pot per treatment was prepared for each type of soil, but no ryegrass was grown. The pot received only the first load of the abovementioned nutrient solution and was then placed in the same growth chamber as the growth trial. Distilled water was supplied weekly to adjust the soil moisture at 80% water-holding capacity to keep the soil moisture in the same range as for the plant trial. The soil incubation lasted until the fourth cut of the ryegrass (i.e., 48 days).

After the incubation, 4 beakers, each containing about 50 g of incubated soils (dry weight equivalent), were prepared for each incubated pot, to make 4 replicates. Following the protocol described in Hooda et al. (1999), the soils were watered to 100% water-holding capacity and equilibrated in an incubator at 24°C for 24 h. After that, the DGT sampler (DGT Research Ltd, Lancaster, UK) was deployed onto the soil surface and incubated at 24°C for 72 h. Then the DGT sampler was dismantled to remove the resin, from which Zn was eluted with 1 ml of 1 M HNO_3_ for 24 h (i.e., DGT extracts).

### Sampling and Analyses

At each cut, the ryegrass shoots were oven-dried at 65°C for 48 h, weighed, and ground using a bead mill equipped with tungsten carbide cups. Subsamples of the plant material were then digested with 4 ml of 8 M HNO_3_ in a high-pressure single-reaction microwave chamber (turboWave, MWS GmbH, Heerbrugg, Switzerland). The same digestion was applied to the dried fertilizers. An aliquot of the digest was purified to extract Zn from the sample matrix. Sample purification was shown to be necessary for Zn isotope analysis with quadrupole single-collector (Q-)ICP-MS in stable Zn isotope soil labeling experiments (Dürr-Auster et al., 2019). A detailed protocol on the separation of Zn from the sample matrix using ion exchange chromatography is provided in Dürr-Auster et al. (2019). In this study, the protocol was applied for plant digests and DGT extracts. The concentration of total Zn was determined for the plant and the fertilizer digests by ICP-OES (ICPE-9820 Series, Shimadzu, Kyoto, Japan). The ^67^Zn:^66^Zn isotope ratio was determined using (Q-)ICP-MS (7500ce, Agilent Technologies, Santa Clara, California, USA) for the purified samples and corrected for mass bias using standard sample bracketing, as explained in Dürr-Auster et al. (2019).

### Data Processing and Analysis

For each cut of each pot, the Zn uptake in the ryegrass shoots at cut *i* [*Zn*_i_ (μg kg^−1^ soil), with *i* varying from 1 to 4] was obtained from the Zn concentration [*CZn*_i_ (μg g^−1^ DW)] and the dry weight [*DW*_i_ (g kg^−1^ soil)] of the corresponding cut:


(1) $$\begin{array}{l} QZn_{i}=CZn_{\text{i}}\times DW_{\text{i}} \end{array}$$

*QZn*_i_ was normalized to 1 kg of soil to describe the transfer of Zn from the soil. The values of *DW*_i_, *CZn*_i_, and *QZn*_i_ in the ryegrass shoot at each cut are shown in Supplementary Table S1.

*QZn*_i_ was composed of the uptake of Zn derived from the fertilizer [*QZndffert*_i_ (μg kg^−1^ soil)], soil [*QZndfsoil*_i_ (μg kg^−1^ soil)], and seeds [*QZndfseed*_i_ (μg kg^−1^ soil)]:


(2) $$\begin{array}{l} QZn_{i}=QZndffert_{\text{i}}+QZndfsoil_{\text{i}}+QZndfseed_{\text{i}} \end{array}$$

In the ryegrass shoot, the relative proportion of Zn derived from the fertilizer [*Zndffert*_i_ (%)], soil [*Zndfsoil*_i_ (%)], and seed [*Zndfseed*_i_ (%)]add up to 100%:


(3) $$\begin{array}{l} 100\%=Zndffert_{\text{i}}+Zndfsoil_{\text{i}}+Zndfseed_{\text{i}} \end{array}$$

The isotopically exchangeable Zn in the soils was labeled with ^67^Zn isotope, and therefore, it had a higher ^67^Zn:^66^Zn ratio than the Zn derived from the fertilizers and seeds. As shown in Supplementary Figure S1, the isotope ratios of ^67^Zn:^66^Zn were in all treatments lower at cut 1 than at the other cuts. In the no Zn treatment, Zn in the shoot was derived solely from the soil or the seed, where the soil was labeled with ^67^Zn (high ^67^Zn:^66^Zn isotope ratio) and the seed was not labeled (low ^67^Zn:^66^Zn isotope ratio). Hence, the lower ^67^Zn:^66^Zn isotope ratios in the shoots at the 1 compared with cuts 2–4 can only derive from a higher contribution of seed Zn to the plant shoot at cut 1 compared with cuts 2–4. For the treatments applied with the Zn-containing fertilizers, the differences between cut 1 and the other cuts were similar to the no Zn treatment. Meanwhile, no significant differences were observed among cuts 2, 3, and 4, regardless of the soils or treatments. Taken together, these results suggest that *QZndfseed* was only significant in cut 1 and was the same among treatments in each soil.

For the no Zn treatment, only two sources of Zn existed, which were soil Zn and seed Zn. Therefore, *Zndfseed* was calculated for each soil on cut 1 of the no Zn treatment as follows [equation adapted from McBeath et al. (2013)]:


(4) $$\begin{array}{l} Zndfseed\\ =\frac{{\;}^{66}Zn_{\text{soil}}\;\cdot \;R_{1\text{\_noZn}}-{\;}^{67}{Zn}_{\text{soil}}}{\left({\;}^{67}{Zn_{\text{seed}}-}^{67}Zn_{\text{soil}}\right)-R_{1\text{\_noZn}}\;\cdot \;\left({\;}^{66}{Zn_{\text{seed}}-}^{66}Zn_{\text{soil}}\right)}\times 100\% \end{array}$$

Where *R*_1_noZn_ refers to the ^67^Zn:^66^Zn (mole mole^−1^) ratio measured in the shoot of cut 1 of the no Zn treatment, ^66^Zn and ^67^Zn refer to the mole fraction of the corresponding isotope (i.e., isotope abundance), whereas the subscript *soil* and *seed* designate values of Zn isotope abundance of the soil and the seed, respectively. *Zn*_soil_ was provided by the shoot at cut 4 of the no Zn treatment. With the successive cuts, seed Zn, if any was left after cut 1, could be continuously translocated to the shoots. Therefore, the more cuts were carried out, the less seed Zn would be left in the soil. The last cut (i.e., cut 4) was used to ensure that the seed Zn was largely exhausted through the previous 3 cuts. *Zn*_seed_ was assumed to be a natural isotope composition (Meija et al., 2016).

For the fertilized treatments, the proportion of Zn derived from the fertilizer and seed (*Zndf* [*fert* + *seed*]_i_ [%]) at cut *i* was calculated, and *Zndfsoil*_i_ was then obtained:


(5) $$\begin{array}{l} {Zndf\left[fert+seed\right]}_{\text{i}}\\ =\frac{{\;}^{66}Zn_{\text{soil}}\;\cdot \;R_{\text{i}}-{\;}^{67}{Zn}_{\text{soil}}}{\left({\;}^{67}{Zn_{\text{fert}}-}^{67}Zn_{\text{soil}}\right)-R_{\text{i}}\;\cdot \;\left({\;}^{66}{Zn_{\text{fert}}-}^{66}Zn_{\text{soil}}\right)}\times 100\% \end{array}$$

(6) $$\begin{array}{l} Zndfsoil_{\text{i}}=100\%-\;{Zndf\left[fert+seed\right]}_{\text{i}} \end{array}$$

Where *R*_i_ refers to ^67^Zn:^66^Zn (mole mole^−1^) measured in the shoot at cut *i* and the subscript *fert* designates values of Zn isotope abundance of the fertilizer, which was deemed to be not distinguishable from the values of seed Zn. The isotope compositions of *Zn*_fert_, *Zn*_soil_, and *Zn*_seed_ are shown in Supplementary Table S2.

For each soil, *QZndfseed* was calculated from the uptake of Zn at cut 1 of the no Zn treatment (*QZn*_1_noZn_). *QZndffert*_1_ was then corrected for seed Zn with the mean value of *QZndfseed* in the same soil:


(7) $$\begin{array}{l} QZndfseed=QZn_{1\text{\_noZn}}\times Zndfseed \end{array}$$

(8) $$\begin{array}{l} QZndffert_{1}=QZn_{1}{\times Zndf\left[fert+seed\right]}_{1}-\bar{QZndfseed} \end{array}$$

*Zndffert*_1_ was calculated from *QZndffert*_1_, while *Zndffert*_2−4_ were hypothesized to be equal to *Zndf* [*fert* + *seed*]_2−4_:


(9) $$\begin{array}{l} Zndffert_{1}=\left({QZndffert}_{1}/QZn_{1}\right)\times 100\% \end{array}$$

(10) $$\begin{array}{l} Zndffert_{2-4}={Zndf\left[fert+seed\right]}_{2-4} \end{array}$$

The values of *Zndffert*_i_ and *Zndfsoil*_i_ in the ryegrass shoot at each cut and *Zndfseed* at cut 1 and cuts 2–4 are shown in Supplementary Table S3.

*QZndffert*_i_ and *QZndfsoil*_i_ in the ryegrass shoots at cut *i* were then calculated:


(11) $$\begin{array}{l} QZndffert_{\text{i}}=QZn_{\text{i}}{\times Zndffert}_{\text{i}} \end{array}$$

(12) $$\begin{array}{l} QZndfsoil_{\text{i}}=QZn_{\text{i}}{\times Zndfsoil}_{\text{i}} \end{array}$$

The values of *QZndfseed*, *QZndffert*_i_, and *QZndfsoil*_i_ in the ryegrass shoot at each cut are shown in Supplementary Table S4.

The recovery of Zn from the fertilizer [*ZnRec*_i_ (%)] in the ryegrass shoot at cut *i* was calculated using:


(13) $$\begin{array}{l} ZnRec_{\text{i}}=\left({QZndffert}_{\text{i}}/QZn_{\text{input}}\right)\times 100\% \end{array}$$

Where *QZn*_input_ is the input of Zn into the soil with the Zn-containing fertilizers (Table 2). The values of *ZnRec*_i_ in the ryegrass shoot at each cut are shown in Supplementary Table S5.

In this paper, the total dry weight [*DW*_sum_ (g kg^−1^ soil)], total Zn uptake [*QZn*_sum_ (μg kg^−1^ soil)], and averaged Zn concentration [*CZn*_avg_ (μg g^−1^ DW)] in the ryegrass shoots of the 4 cuts are discussed. They were calculated for each pot, as follows:


(14) $$\begin{array}{l} DW_{\text{sum}}=DW_{1}+DW_{2}+DW_{3}+DW_{4} \end{array}$$

(15) $$\begin{array}{l} QZn_{\text{sum}}=QZn_{1}+QZn_{2}+QZn_{3}+QZn_{4} \end{array}$$

(16) $$\begin{array}{l} CZn_{\text{avg}}=\left({CZn}_{1}+CZn_{2}+CZn_{3}+CZn_{4}\right)/4 \end{array}$$

Then, the total uptake and averaged proportion of Zn derived from the fertilizer [*QZndffert*_sum_ (μg kg^−1^ soil); *Zndffert*_avg_ (%)] and the soil [*QZndfsoil*_sum_ (μg kg^−1^ soil); *Zndfsoil*_avg_ (%)] of the 4 cuts were calculated:


(17) $$\begin{array}{l} QZndffert_{\text{sum}}=QZndffert_{1}+QZndffert_{2}+QZndffert_{3}\\ \;+QZndffert_{4} \end{array}$$

(18) $$\begin{array}{l} QZndfsoil_{\text{sum}}=QZndfsoil_{1}+QZndfsoil_{2}+QZndfsoil_{3}\\ +QZndfsoil_{4} \end{array}$$

(19) $$\begin{array}{l} Zndffert_{\text{avg}}=(Zndffert_{1}+Zndffert_{2}+Zndffert_{3}\\ \;+Zndffert_{4})/4 \end{array}$$

(20) $$\begin{array}{l} Zndfsoil_{\text{avg}}=(Zndfsoil_{1}+Zndfsoil_{2}+Zndfsoil_{3}\\ \;+Zndfsoil_{4})/4 \end{array}$$

The total recovery of Zn from the fertilizer [*ZnRec*_sum_ (%)] in the ryegrass shoot at cuts 1–4 was calculated:


(21) $$\begin{array}{l} ZnRec_{\text{sum}}=\left({QZndffert}_{\text{sum}}/QZn_{\text{input}}\right)\times 100\% \end{array}$$

The apparent fertilizer use efficiency [*AUE* (%)] was also calculated to compare the results to *ZnRec*_sum_, which relies only on the relative increase of *QZn*_sum_ resulting from the addition of Zn with the fertilizer:


(22) $$\begin{array}{l} AUE=\frac{QZn_{\text{sum\_fert}}-{\bar{QZn}}_{\text{sum\_noZn}}}{QZn_{\text{input}}}\times 100\% \end{array}$$

Where *QZn*_sum_fert_ and *QZn*_sum_noZn_ are respectively the Zn uptake in the ryegrass shoots (*QZn*_sum_) with and without Zn fertilization in the same soil. For *QZn*_sum_noZn_, the mean value of the replicates was used.

### Statistical Analysis

Data were processed using R 3.6.3 statistical software. Identification of significant differences among treatments in the plant growth trial was performed with analysis of variance (ANOVA) and Tukey's honest significant difference *post hoc* multiple comparison. Two-way ANOVA was performed to test the significance of soil effects, fertilizer effects, and their interaction in the plant growth trial. The Bland–Altman approach was used to compare the Zn recovery calculated with stable isotope technique and the AUE method and, to compare the ^67^Zn:^66^Zn isotope ratios, and also the proportion of Zn derived from the fertilizers, measured with the DGT extracts and the plants. This approach allows agreements or discrepancies between two analytical methods to be assessed by illustrating the bias and 95% confidence interval of the absolute difference between the values obtained by the two methods (Bland and Altman, 1986).

## Results

### Plant Growth and Zn Accumulation

The addition of complex organic fertilizers to the two soils had distinct effects on shoot dry weight, Zn concentration, and Zn uptake (Table 3). In the Heitenried soil, the addition of organic fertilizers had no impact on shoot Zn concentrations. However, the shoot dry weight and Zn uptake increased by up to factor 1.2 with the addition of poultry manure, compared with those in the Heitenried soil that received no Zn. In the Strickhof soil, by contrast, no significant impact of the Zn-fertilizer additions was observed on the production of shoot dry matter. However, the addition of poultry and cattle manure increased the Zn concentration in the shoots by 10–20%, compared with the no Zn treatment in the Strickhof soil. The highest shoot Zn concentration was observed in the cattle manure, followed by the poultry manure treatment, and the same hierarchy was found for the Zn uptake. A direct comparison of both soils revealed that the shoot dry weight was higher, whereas the Zn concentration and Zn uptake were lower in the Strickhof soil than in the Heitenried soil (Table 3).

**Table 3:** Total dry weight (DW), averaged Zn concentration, total Zn uptake, and averaged proportion of Zn derived from the Zn-containing fertilizers (*Zndffert*_avg_%) in the Italian ryegrass shoot (sum of 4 cuts) cultivated in a growth chamber and grown in Heitenried soil (pH = 4.9) and Strickhof soil (pH = 7.7) amended with distinct Zn-containing fertilizers.

**Soil**	**Treatment**	**Shoot dry weight**	**Zn concentration in shoots**	**Zn uptake in shoots**	***Zndffert*_avg_**
		**g DW kg^**1**^ soil**	**μg Zn g^**−1**^ DW shoots**	**μg Zn kg^**1**^ soil**	**% of Zn uptake**
*Heitenried*	No Zn	13.1 ± 1.9 a	54.9 ± 1.6 a	719 ± 99 a	–
	ZnSO_4_	14.6 ± 0.6 ab	56.4 ± 1.0 a	816 ± 29 ab	16.9 ± 0.8 a
	Sewage sludge	15.1 ± 0.9 ab	56.3 ± 0.8 a	833 ± 50 ab	19.8 ± 0.4 b
	Poultry manure	15.9 ± 0.9 b	54.5 ± 2.1 a	865 ± 32 b	20.2 ± 0.6 b
	Cattle manure	14.9 ± 0.5 ab	57.3 ± 1.8 a	851 ± 40 b	19.8 ± 0.3 b
*Strickhof*	No Zn	16.3 ± 0.7 a	38.3 ± 1.1 a	621 ± 41 a	-
	ZnSO_4_	16.2 ± 0.4 a	40.6 ± 0.4 bc	656 ± 11 ab	12.1 ± 0.8 a
	Sewage sludge	16.6 ± 0.2 a	39.7 ± 0.4 ab	656 ± 0.9 ab	10.6 ± 0.4 a
	Poultry manure	16.3 ± 0.5 a	42.1 ± 0.9 c	690 ± 25 bc	10.8 ± 1.4 a
	Cattle manure	15.7 ± 1.1 a	46.1 ± 1.3 d	726 ± 31 c	10.7 ± 0.3 a
ANOVA	Soil	***	***	***	***
	Fertilizer	*	***	***	*
	Soil × Fertilizer	*	***	*ns*	***

*Values represent the mean values of n = 4 replicates ± standard deviation. Zndffert_avg_ values are corrected for the uptake of Zn derived from the seed*.

*Zndffert_avg_ was calculated as the average proportion of Zn uptake derived from the fertilizer at cuts 1–4*.

*For each soil, different letters in the same column indicate significant differences (p < 0.05) among treatments*.

Two-way ANOVA was performed to assess the effects of the soil and fertilizer treatment on data in the same column: ns stands for no significance difference whereas *, **, and *** indicate significant differences at the probability level of 0.05, 0.01, and 0.001, respectively.

### Fraction of Plant Zn Derived From Organic Fertilizers, Soils, and Seeds

**Figure 1:**
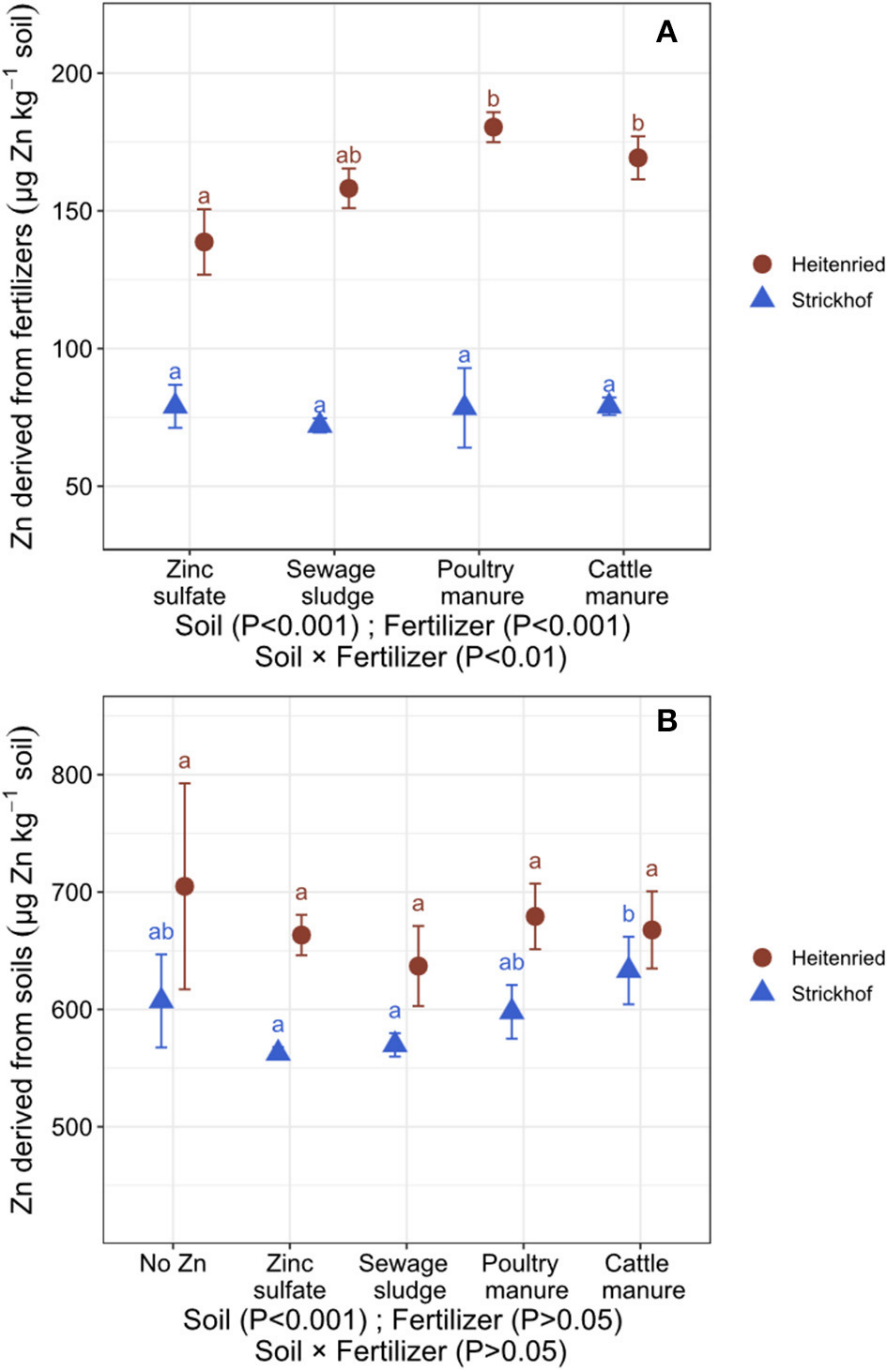
Uptake of Zn derived from the Zn-containing fertilizers (*QZndffert*_sum_; **A**) and soils (*QZndfsoil*_sum_; **B**) in the Italian ryegrass shoots (sum of 4 cuts) cultivated in a growth chamber and grown in soil from Heitenried (pH = 4.9) and Strickhof (pH = 7.7) without (no Zn) and with the addition of distinct Zn-containing fertilizers. Data are average values ± standard deviation (bars in the figures) calculated from *n* = 4 replicates. *QZndffert*_sum_ values are corrected for the uptake of Zn derived from the seed. For each soil, different letters indicate significant differences (*p* < 0.05) among treatments. The effects of the soils and fertilizers tested by two-way ANOVA are shown at the bottom of each plot.

By labeling the soil available Zn pool with ^67^Zn, we could precisely calculate the contribution of Zn derived from organic fertilizers, soils, and seeds to the Zn uptake in the ryegrass shoots. In the Heitenried soil, most of the Zn in the shoots derived from the soil (*Zndfsoil*_avg_; 79–82% for fertilizer treatments) whereas about one fifth derived from the fertilizers (*Zndffert*_avg_; 17–20%; Table 3). *Zndffert*_avg_ and the quantity of Zn that was transferred from the freshly applied fertilizers to the shoots (*QZndffert*_sum_) were the lowest with ZnSO_4_ and highest with the animal manures (Figure 1A). In addition, the quantity of Zn that was transferred from the soil to the ryegrass shoots (*QZndfsoil*_sum_) did not significantly differ among the treatments (Figure 1B). In the Strickhof soil, 86–88% of the plant Zn uptake derived from the soil whereas 11–12% derived from the fertilizers (Table 3, Figure 1). The contribution of Zn derived from freshly applied fertilizer to shoot Zn uptake (*ZNdffert*_avg_ and *QZndffert*_sum_) did not differ between treatments. In addition, *QZndfsoil*_sum_ were not distinguishable between the fertilized and non-fertilized treatments (Figure 1B).

The stable isotope ^67^Zn was further employed to precisely calculate the contribution of Zn from the ryegrass seeds. With the seeds, 44 μg Zn kg^−1^ soil was added to the soils. Given the values of *QZndffert*_sum_ in the ryegrass shoots (72–180 μg Zn kg^−1^ soil), Zn derived from the seed could be an important third source and lead to an overestimated uptake of Zn derived from the fertilizer. In the no Zn treatment, the soil (^67^Zn labeled) and the seeds (non-labeled) were the only sources of Zn for the ryegrass shoots. For the first cut, the ^67^Zn:^66^Zn isotope ratios were significantly lower than for cuts 2–4 in all treatments (Supplementary Figure S1). These results indicate that seed Zn was a substantial source of Zn in the initial growth phase of the ryegrass. Based on the ^67^Zn:^66^Zn isotope ratios in the no Zn treatment (Equation 4), we calculate that the seed Zn contributed to 1.64–2.25% of the total Zn uptake in the ryegrass shoot.

### Comparison of Plant Use Efficiency of Fertilizer Zn Obtained From Mass Balance and Isotopic Approaches

The fate of Zn derived from freshly applied complex organic fertilizers in soil–plant systems can be assessed by calculating the recovery of fertilizer Zn in the plant. The recovery can be calculated with a mass-balance *AUE* approach or with an isotope labeling approach (*ZnRec*_sum_; Table 4). A direct comparison of the two methods revealed that *AUE* (2.3–9.7%) was overall lower than *ZnRec*_sum_ (4.7–12.0%). Meanwhile, the *AUE* values had a higher standard deviation (SD = 0.6–3.3%) than the stable isotope *ZnRec*_sum_ results (SD = 0.2–1.0%). The Bland–Altman plot in Supplementary Figure S2 compares the *AUE* and *ZnRec*_sum_ that were obtained in this study. On average, *AUE* was 1.8% lower than *ZnRec*_sum_. This bias was 16.2% of the total range of Zn recovery calculated with the two methods. For each of the two soils, there was apparently a positive trend between the mean and difference of the results that were obtained with the two approaches. This trend indicates that the bias was not consistent and thus could not be corrected. The limit of agreement was between −6.1 and 2.5% based on the 95% confidence intervals (CI), meaning that *AUE* could be 4.3% below or above *ZnRec*_sum_. Compared with the total range of Zn recovery calculated in this study, the limit of agreement was 55% of the range of all *ZnRec*_sum_ values.

**Table 4:** Recovery of Zn derived from the Zn-containing fertilizers in the Italian ryegrass shoot (sum of 4 cuts) cultivated in a growth chamber and grown in Heitenried soil (pH = 4.9) and Strickhof soil (pH = 7.7) amended with distinct Zn-containing fertilizers, measured with the stable isotope approach (*ZnRec*_sum_) and the AUE approach.

**Soil**	**Treatment**	***ZnRec*_sum_**	***AUE***
		**% of Zn input**	**% of Zn input**
*Heitenried*	ZnSO_4_	9.8 ± 0.8 a	6.8 ± 2.0 a
	Sewage sludge	10.4 ± 0.5 ab	7.5 ± 3.3 a
	Poultry manure	12.0 ± 0.4 b	9.7 ± 2.1 a
	Cattle manure	10.9 ± 0.5 ab	8.5 ± 2.6 a
*Strickhof*	ZnSO_4_	5.6 ± 0.5 a	2.4 ± 0.8 a
	Sewage sludge	4.7 ± 0.2 a	2.3 ± 0.6 a
	Poultry manure	5.2 ± 1.0 a	4.6 ± 1.7 ab
	Cattle manure	5.1 ± 0.2 a	6.7 ± 2.0 b
ANOVA	Soil	***	***
	Fertilizer	*	*
	Soil × Fertilizer	**	*ns*

*Values represent the mean values of n = 4 replicates ± standard deviation. ZnRec_sum_ values are corrected for the uptake of Zn derived from the seed*.

*ZnRec_sum_ was calculated as the uptake of Zn derived from the fertilizer in all four cuts of ryegrass shoots divided by the total input of Zn with the fertilizer*.

*Apparent use efficiency was calculated as the relative increase in the uptake of Zn in all four cuts between the fertilized and non-fertilized ryegrass divided by the total input of Zn with the fertilizer*.

*For each soil, different letters in the same column indicate significant differences (p < 0.05) among treatments*.

Two-way ANOVA was performed to assess the effects of the soil and the fertilizer treatment on data in the same column: ns stands for no significance difference whereas *, **, and *** indicate significant differences at the probability level of 0.05, 0.01, and 0.001, respectively.

### Comparison of Zinc Isotope Ratios and Proportion of Fertilizer Zn in Plants and DGT Extracts

**Figure 2:**
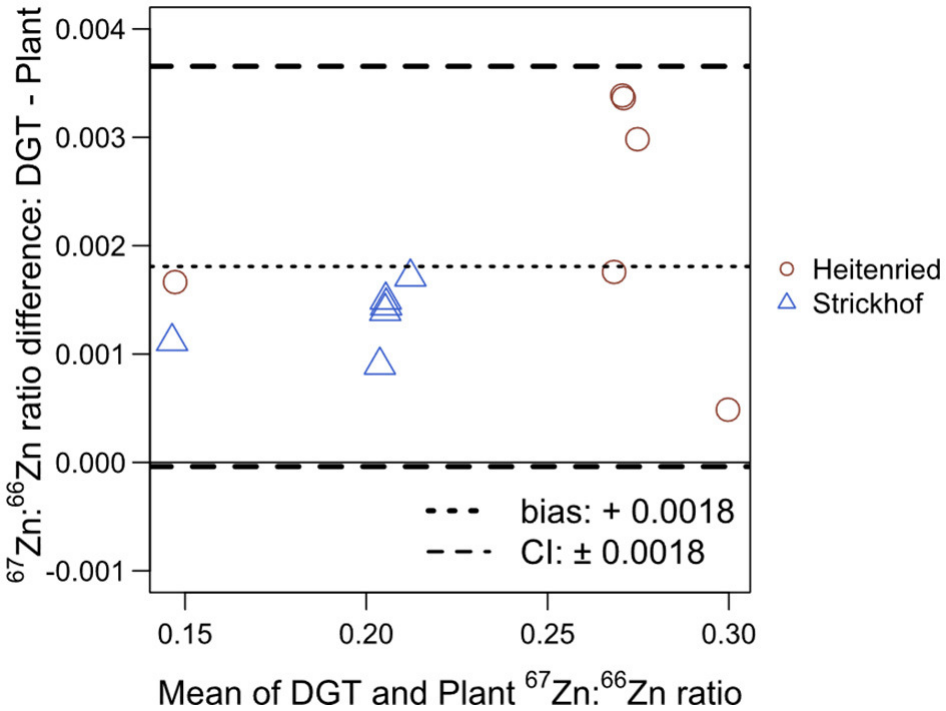
Bland–Altman plot comparing the ^67^Zn:^66^Zn ratios measured in the DGT extracts in Heitenried soil and Strickhof soil incubated for 48 days after the addition of distinct Zn-containing fertilizers and in the Italian ryegrass shoots at cut 4 subjected to the same treatment cultivated in the same soil in the same growth chamber. For each treatment, the results measured in the DGT extracts and the shoots were averaged (x-axis) and plotted against their difference (y-axis). The dotted line shows the bias and the dashed lines represent the 95% confidence interval (CI) of the Bland–Altman analysis.

The plants and the ^67^Zn:^66^Zn isotope ratios measured with the DGT were compared using a Bland–Altman plot (Figure 2). For the plant, only Zn in cut 4 was considered in this comparison, to minimize the influence of seed Zn (Supplementary Figure S1; Table S4). The ^67^Zn:^66^Zn ratios of the DGT extracts were on average 0.0018 units higher than those in the shoots. Meanwhile, there was no obvious relationship between the mean and the difference. The 95% confidence interval was ±0.0018 units, indicating that the ^67^Zn:^66^Zn isotope ratios measured in the DGT extracts can be higher or lower by up to 0.0018 units than those in the shoots without showing any significant statistical difference. Both the bias and the confidence interval account for 1.2% of the range of all the isotope ratios that were measured in this study.

**Figure 3:**
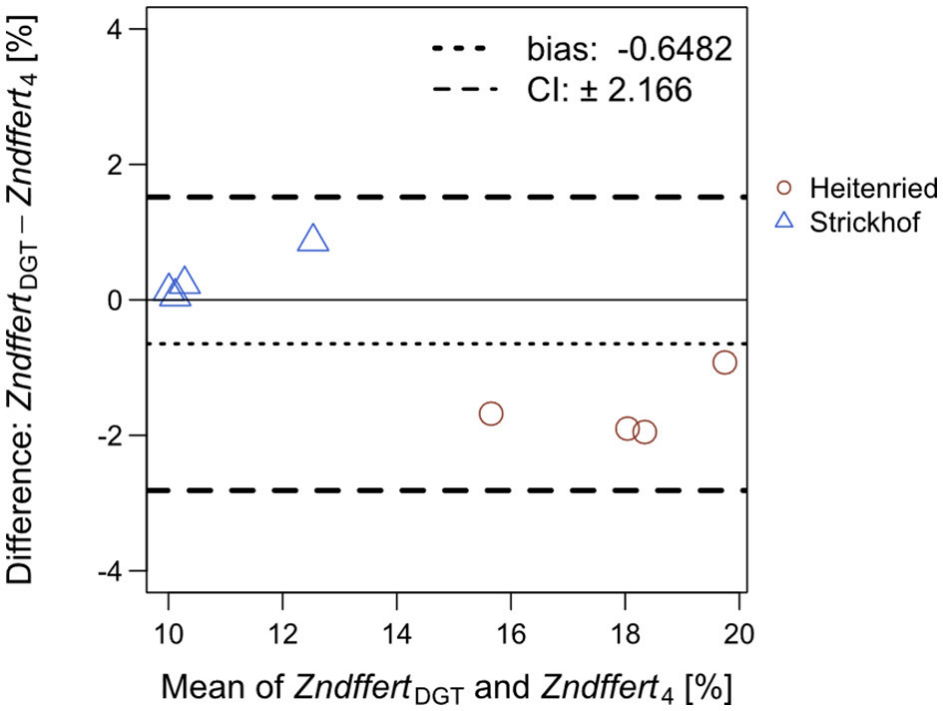
Bland–Altman plot comparing the proportion of Zn derived from the Zn-containing fertilizers in the DGT extracts (*Zndffert*_DGT_) and in Italian ryegrass shoots at cut 4 (*Zndffert*_4_) subjected to the same treatment in the same soil. For each treatment, the values of *Zndffert*_DGT_ and *Zndffert*_4_ were averaged (x-axis) and plotted against their difference (y-axis). The dotted line shows the bias and the dashed lines represent the 95% confidence interval (CI) of the Bland–Altman analysis.

The proportion of DGT-extracted Zn that derived from the fertilizers [*Zndffert*_DGT_ (%)] was calculated following the same principles as for the plant (Equation 5). In the equation, ^67^Zn:^66^Zn ratios measured in the DGT extracts were used instead of those in the plant, whereas the soil Zn isotope abundances of ^67^Zn and ^66^Zn were measured in the DGT extracts in the no Zn treatment instead of the abundances measured in the ryegrass shoots. In Figure 3, *Zndffert*_DGT_ values were compared with *Zndffert*_4_ values measured in the ryegrass shoots at cut 4 (Supplementary Table S3) through a Bland–Altman plot. *Zndffert*_DGT_ were on average 0.6% lower than *Zndffert*_4_, which accounts for 6% of the total range of *Zndffert*_DGT_ and *Zndffert*_4_ values (10–20%). Meanwhile, *Zndffert*_DGT_ and *Zndffert*_4_ were highly positively correlated in each of the soils (*p* < 0.05) and in both soils (*R* = 0.987; *p* < 0.001; Supplementary Figure S3).

## Discussion

### Contribution of Complex Organic Fertilizers to the Zinc Nutrition of Ryegrass

In both soils, the addition of water-soluble Zn as ZnSO_4_ did not alter the shoot dry weight (*DW*_sum_), compared with the no Zn treatment (Table 3). These results suggest that the initial availability of Zn in the soil did not limit the plant biomass production. The Zn concentrations in the ryegrass shoots in the no Zn and ZnSO_4_ treatments ranged from 38 to 56 μg Zn g^−1^ DW, which indicates that the plants were neither Zn limited nor exposed to excess Zn (Broadley et al., 2007).

The higher soil pH in the alkaline Strickhof soil (pH 7.7) may have caused the overall higher shoot dry weight, lower Zn concentration and uptake, and lower *QZndffert*_sum_ and *QZndfsoil*_sum_ than those in the acidic Heitenried soil (pH 4.9). For the higher shoot dry weight in the Strickhof soil, one explanation would be that the growth of Italian ryegrass was favored under neutral to alkaline soil conditions (Gregory and Nortcliff, 2013; CABI, 2020). The availability of Zn is higher in acidic than in alkaline soils, but also other soil properties such as total Zn concentration and soil carbonate concentration can affect Zn availability (Mertens and Smolders, 2013). The *QZndffert*_sum_ and total Zn uptake were higher in the acidic Heitenried compared with the alkaline Strickhof soil (Table 3). These results suggest that soil Zn had a minor impact on Zn plant uptake, since soil Zn was about two times as high in the Strickhof compared with the Heitenried soil (Table 1). The higher soil pH, and likely the higher carbonate concentration, in the Strickhof soil may have reduced the solubility of Zn derived from the fertilizer and soil due to a stronger adsorption of Zn on negatively charged binding sites of the soil (Diesing et al., 2008; Imseng et al., 2019). The lower solubility of fertilizer Zn and soil Zn in the alkaline Strickhof soil may have further lowered the concentration and uptake of Zn in the ryegrass shoots.

The animal manures significantly increased the plant uptake of Zn, compared with the no Zn treatment in both soils (Table 3). Our results show that the enhanced Zn uptake was related to higher dry matter production in the acidic Heitenried soil and to an increased shoot Zn concentration in the alkaline Strickhof soil. In the Heitenried soil, *Zndffert*_avg_ and *QZndffert*_sum_ were significantly higher with the application of the animal manures than with ZnSO_4_ (Table 3, Figure 1A). Since very similar quantities of Zn were added to the soils among the different fertilization treatments, these results suggest that the Zn in the animal manures was more available to the ryegrass than ZnSO_4_ over one growth cycle. However, the distinct Zn availabilities among the fertilizers did increase neither the Zn concentration nor the Zn uptake into the ryegrass (Table 3). Unlike in the Heitenried soil, some of the fertilizers increased the shoot Zn concentrations in the alkaline Strickhof soil (Table 3). The fertilization treatments had no significant effects on *Zndffert*_avg_ and *QZndffert*_sum_, which indicates similar availability of Zn derived from these fertilizers to the ryegrass in the Strickhof soil. These results suggest that the increased Zn concentration and/or Zn uptake in the ryegrass shoots with the addition of ZnSO_4_ and the animal manures are likely induced through the Zn addition with these fertilizers.

The addition of green manure to alkaline soils can solubilize Zn in the soil pool and increase the transfer of soil Zn to the plant (Aghili et al., 2014). This increase has been ascribed to changes in soil pH or water-soluble organic ligands that increase the solubility of Zn in the soils during the decomposition of green manures (Gramlich et al., 2013; Soltani et al., 2014). In this study, the Zn, C, and N inputs with the organic fertilizers (Table 2) were similar to those in Aghili et al. (2014). However, *QZndfsoil*_sum_ did not differ between the fertilized and nonfertilized treatments (Figure 1B), indicating that a Zn solubilization in alkaline soils through organic fertilizer may depend on soil and fertilizer properties. The alkaline Strickhof soil contained about 100 times more CaCO_3_ compared with the soil used by Aghili et al. (2014; Table 1). The higher CaCO_3_ concentration in the Strickhof soil may have buffered soil acidification effects caused by the nitrification processes and bicarbonates production during fertilizer decomposition. In addition, the DTPA-extractable Zn in the soil used by Aghili et al. (2014) was around 10 times lower than in the soils used in this study (Table 1). Hence, in this study, water soluble ligands such as amino acids that were potentially produced during the decomposition of the fertilizers (Soltani et al., 2014) may have rather formed complexes with Zn that was already in the solution than with Zn that was tightly bound to the soil matrix.

Moreover, the main components of the green manure used by Aghili et al. (2014) and the organic fertilizer used in our study differed. Green manure contains fresh plant material whereas the complex organic fertilizers contain feces, inorganic components, and, in the animal manures, undigested plant residues (e.g., mature lignified plant material; Rose et al., 2015). These distinct forms of organic matter influence the decomposition rate (Thuriès et al., 2001) and decomposition products of the organic fertilizers (Ohno and Crannell, 1996), and therefore also likely the soil properties that control the solubility of Zn. Unlike animal manure, green manure added to a soil might quickly release low-molecular weight ligands, which leads to a desorption of Zn from the soil surface (Ohno and Crannell, 1996; Thuriès et al., 2001; Soltani et al., 2014). Furthermore, in this study, the duration of the plant growth trial in the fertilized soils was 48 days which is much shorter than the 125 days in Aghili et al. (2014). In soils incubated with complex organic fertilizers, the decomposition of organic fertilizers could last for more than 3 months and result in reduced soil pH and increased soil Zn availability after 60 days of incubation (Tella et al., 2016). Together, an increase of the available Zn in alkaline soils upon the addition of organic fertilizer may depend on soil and fertilizer properties such as buffer capacity and the main components of organic fertilizer, and also the incubation time for the fertilizer in soils. Clearly, further studies are needed to identify major mechanisms and types of organic fertilizer that solubilize Zn in alkaline soils.

The result that 11–20% of the Zn in the plant derived from the fertilizers is at the lower end of the scale, compared with previous observations (Amer et al., 1980; McBeath et al., 2013; Aghili et al., 2014; McBeath and McLaughlin, 2014; Mattiello et al., 2021). Similar source-tracing studies revealed that *Zndffert*_avg_ values ranged from 0.1 to 99% with a median of 19% (Dürr-Auster et al., 2019). The *Zndffert*_avg_ values are mainly related to the soil properties, Zn solubility, and quantity of Zn applied. The values >50% are reported for the high input of water-soluble Zn or Zn oxides into sandy and calcareous soils, whereby the fertilizer Zn represents a major available source for plant Zn uptake (McBeath and McLaughlin, 2014). Lower values (<50%) are either reported for soils with relatively small and large Zn available pools that received fertilizers with which Zn cannot be easily dissolved in soils, including Zn-coated fertilizers and organic fertilizers (e.g., plant residues; McBeath et al., 2013; Aghili et al., 2014; Dürr-Auster et al., 2019; Mattiello et al., 2021). The isotope results of our study indicated that the Zn derived from the complex organic fertilizers was equal or more available to the ryegrass than the soluble ZnSO_4_. Hence, the low *Zndffert*_avg_ values found in this study might not be related to the low solubility of Zn in the complex organic fertilizers. As an alternative explanation, the DTPA-extractable Zn in the soil, which represents the soil available Zn pool, was 2.6–3.7 times higher than the Zn added with fertilizers (Tables 1, 2). This comparison indicates that the Zn added with the fertilizers was diluted with the soil available Zn pool, which could explain the comparably low contribution of Zn applied with fertilizer to plant Zn.

### Most Zinc Freshly Applied With Complex Organic Fertilizers Remains in the Soil

The relatively high confidence limits between *AUE* and *ZnRec*_sum_ and the positive trend shown in the Bland–Altman plot (Supplementary Figure S2) indicate that the non-isotope AUE approach cannot be used interchangeably with the isotope approach. Meanwhile, the high standard deviation of *AUE* (Table 4) illustrates that the stable isotope source-tracing technique is more precise than the AUE approach.

Previous studies have shown that complex organic fertilizer can be a main input into soils and potentially contribute to critical soil Zn accumulation. For example, in the Broadbalk long-term experiment, the concentration of Zn in the topsoil significantly increased with the application of farmyard manure over 160 years, as compared with the plot where no fertilizers were applied (Fan et al., 2008). Similarly, by determining the Zn fluxes at three Swiss meadow sites, animal manure application was recognized as the main Zn input into soils and significantly contributed to the current soil Zn accumulation in these grassland systems (Imseng et al., 2019). Such soil Zn accumulation could pose a threat to soil ecosystems, considering the potential toxicity of Zn on plants, invertebrates, and microbes (Påhlsson, 1989; Wang et al., 2007; Santorufo et al., 2012). The isotope results presented here provide complementary data to these soil Zn mass balances. The *ZnRec*_sum_ values ranged from 4.7 to 12.0% (Table 4), indicating that only a minor fraction of Zn derived from freshly applied fertilizer was transferred to the ryegrass shoots. Consequently, the majority of the fertilizer derived Zn remained in the soil after one crop cycle (~90%) and could be available for the next crop cycles and/or contribute to the buildup of a residual pool of Zn in a long run. Nevertheless, it is noteworthy that in this study, the plant growth trial lasted 48 days, and the results might be more representative of short crop cycles. As mentioned above, organic fertilizers could continuously decompose in soils for a longer time period, during which the desorption of Zn from Fe oxides in organic fertilizers could release available Zn to plants (Tella et al., 2016). Moreover, the quantity of the soil used for ryegrass cultivation was small, and the light intensity in the growth chamber was low, relative to those in the field. These conditions might limit the plant growth and total Zn uptake due to the pot-bound root system and the depressed photosynthesis efficiency (Poorter et al., 2012) and result in an underestimated *ZnRec*_sum_. Hence, the plant use efficiency of Zn derived from complex organic fertilizers remains to be tested for longer crop cycles, such as on perennial crops, under field conditions.

### Can DGT Be Used as a Proxy for Assessing the Proportion of Zn Derived From Fertilizers in Plants?

Diffusive gradients in thin films technique is an established technique to assess the availability of soil Zn to plants (Cornu and Denaix, 2006; Degryse et al., 2009; Tandy et al., 2011). Here, we aimed to expand the scope of DGT by measuring isotope ratios in DGT extracts and thereby determine the contribution of soil and fertilizer Zn to the soluble Zn fraction in the soils. As shown in Figures 2 and 3, very similar isotope ratios of ^67^Zn:^66^Zn and *Zndffert* values were obtained in the DGT extracts and ryegrass shoots, revealing that the DGT and plant extracted Zn from the same pool in the soils. Hence, *Zndffert*_DGT_ revealed the distinct impacts of Zn-containing fertilizers and soil pH on the contribution of fertilizer Zn to the soil available Zn pool, which are the same as those investigated through the plant trial. These results suggest that, instead of ryegrass, DGT can be adequately employed to predict the relative contribution of fertilizer and soil sources to the available Zn pool in soils, which is energy- and labor-saving as compared with plant cultivation. To our knowledge, this is the first application of DGT for Zn source tracing in soil-fertilizer systems.

## Conclusion

Our study demonstrated that stable Zn isotope soil labeling can be used to precisely determine the contribution of complex organic fertilizer to the Zn nutrition of plants and to precisely determine the fate of Zn applied with fertilizer in soil–plant systems. Particularly, we showed that the largest fraction of Zn in the ryegrass shoot was derived from the soil (79–88%), followed by the Zn-containing fertilizer (11–20%), and the seed (<2.3%) after 48 days of growth. The plant recovery of Zn derived from freshly applied complex organic fertilizers was low (4.7–12%), whereas the major fraction of fertilizer Zn remained in the soil (>88%). These findings illustrate that complex organic fertilizer may contribute to the buildup of a Zn residual pool in the soil. In both the acidic Heitenried soil and the alkaline Strickhof, animal manures increased the plant uptake of Zn more effectively than the water-soluble ZnSO_4_. Meanwhile, our results indicate that Zn in the animal manure has an equal or even higher availability than ZnSO_4_ for the ryegrass grown in the two soils. Finally, this study indicates that the passive DGT sampler extracted Zn from the same soil pool as the ryegrass. The scope of DGT could be therefore extended to estimate the relative contribution of Zn fertilizers to the soil available Zn pool and therefore to plant nutrition.

## Data Availability Statement

The original contributions presented in the study are included in the article/Supplementary Materials, further inquiries can be directed to the corresponding authors.

## Author Contributions

B-FY wrote original draft, formal analyses, data curation, and visualization. TD-A involved in methodology, resources, validation, experiments, and data collection. EF conducted conceptualization, methodology, validation, data curation, writing review and editing, and funding acquisition. MW contributed to review and editing the manuscript and to investigations. All authors contributed to the article and approved the submitted version.

## Funding

This study was funded by the Swiss State Secretariat for Education and Research within the framework of the COST (European Cooperation for Science and Technology) action Mineral-Improved Crop Production for Healthy Food and Feed (FA0905) with the Project Number C10.0085 and by the Group of Plant Nutrition.

## Conflict of Interest

The authors declare that the research was conducted in the absence of any commercial or financial relationships that could be construed as a potential conflict of interest.

## Publisher's Note

All claims expressed in this article are solely those of the authors and do not necessarily represent those of their affiliated organizations, or those of the publisher, the editors and the reviewers. Any product that may be evaluated in this article, or claim that may be made by its manufacturer, is not guaranteed or endorsed by the publisher.
