# Supplementary material for: The Use of Stable Zinc Isotope Soil Labeling to Assess the Contribution of Complex Organic Fertilizers to the Zinc Nutrition of Ryegrass
Source: Front Plant Sci. 2021 Dec 21;12:730679. doi: 10.3389/fpls.2021.730679 (PMC8724203; doi:10.3389/fpls.2021.730679)
Supplement: Supplementary file 1 [file Data_Sheet_1.docx]

**Supporting information for:**

**The use of stable zinc isotope soil labeling to assess the contribution of complex organic fertilizers to the zinc nutrition of ryegrass**

Bo-Fang Yan^1,†,*^, Thilo Dürr-Auster^1,†^, Emmanuel Frossard^1^, Matthias Wiggenhauser^1,**^

^1^ Department of Environmental Systems Science, Group of Plant Nutrition, ETH Zurich, Eschikon 33, CH-8315 Lindau, Switzerland

^†^These authors have contributed equally to this work

^*^Corresponding author: Email address: zansybf@163.com

^**^Corresponding author: Email address: matthias.wiggenhauser@usys.ethz.ch


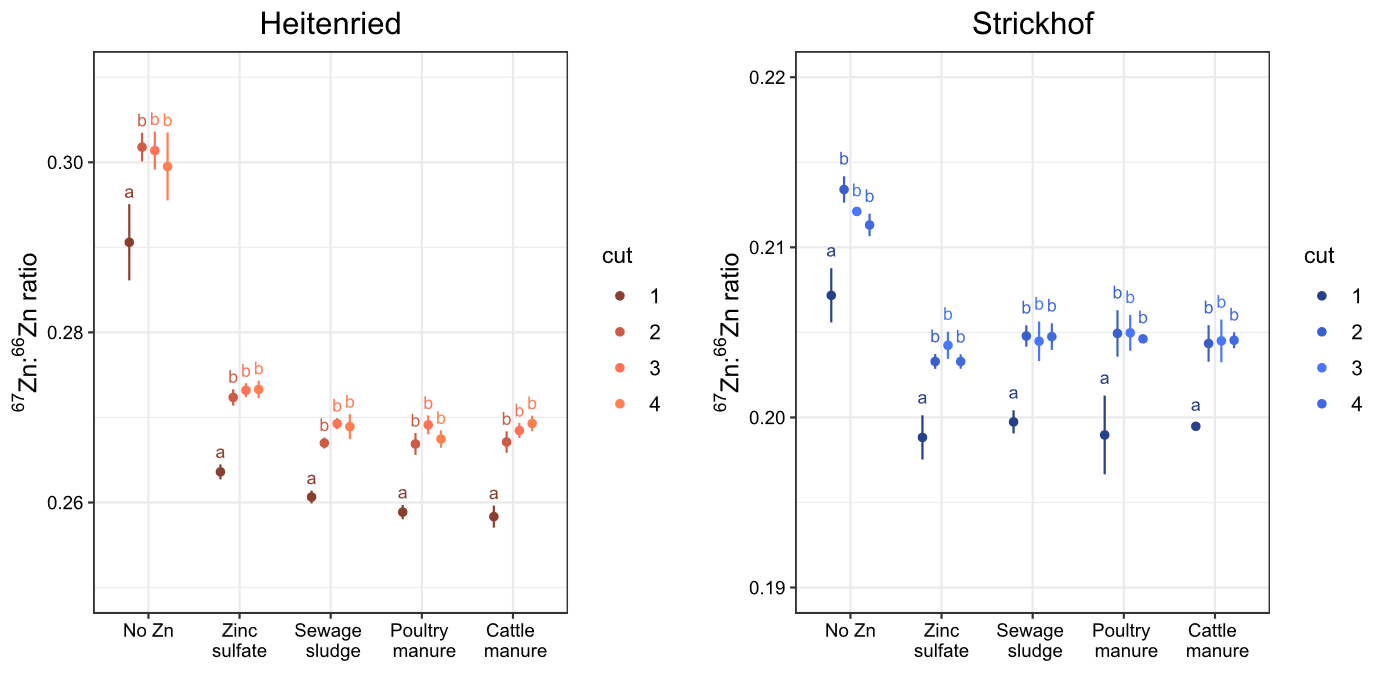


**Supplementary Figure S1.** ^67^Zn:^66^Zn ratios of ryegrass shoots cultivated in a growth chamber and grown in Heitenried soil (pH = 4.9, left) and Strickhof soil (pH = 7.7, right) amended with distinct Zn-containing fertilizers measured at different cuts. Data are average values ± standard deviation (bars in the figures) calculated from *n* = 4 replicates. Different letters indicate the significant difference between the cuts with the respective treatments (*P* <0.05).


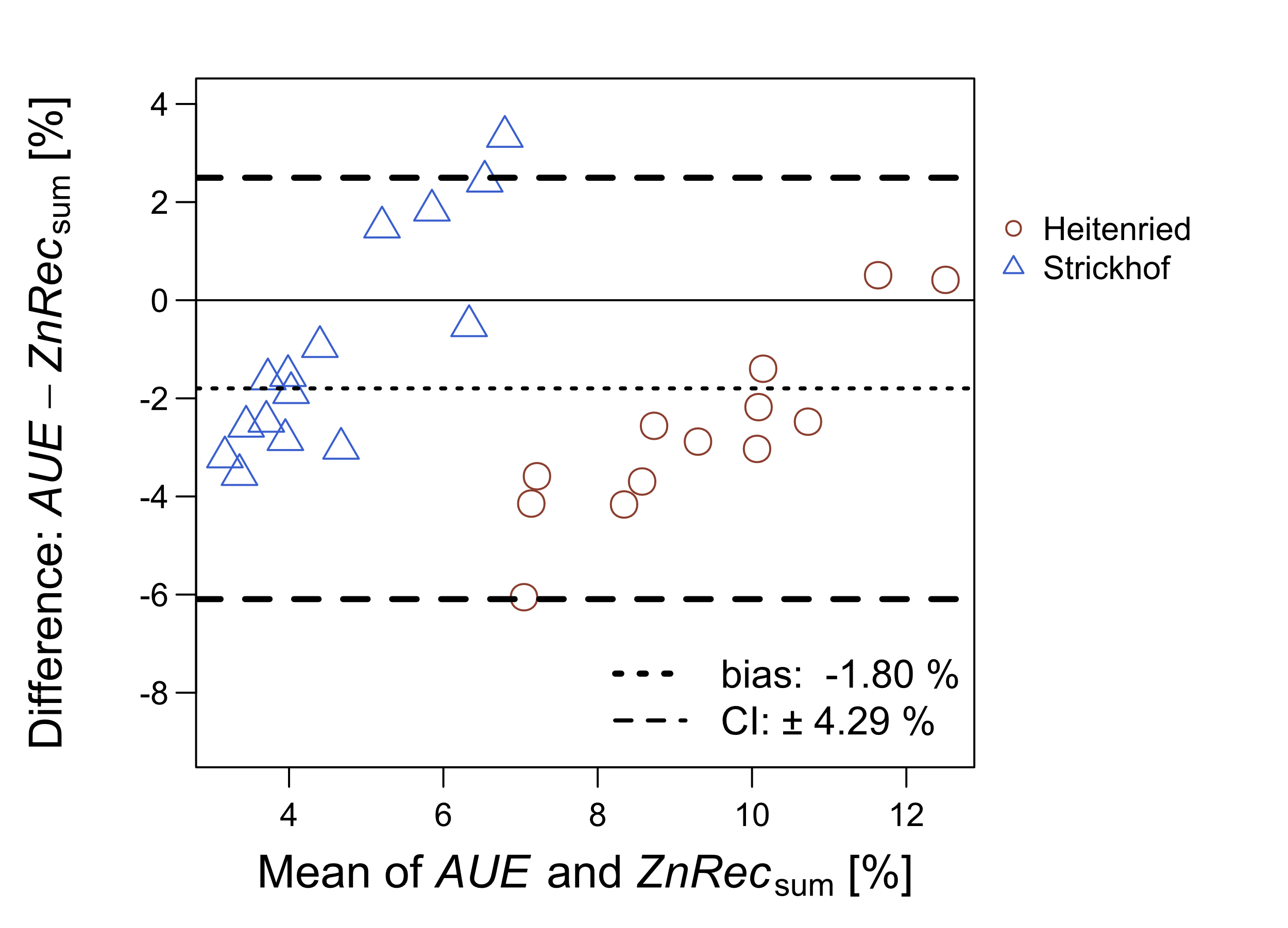


**Supplementary Figure S2.** Bland–Altman plot comparing the recovery of Zn derived from the Zn-containing fertilizers in the Italian ryegrass shoots (sum of 4 cuts) grown in the same pot measured with the stable isotope approach (*ZnRec*_sum_) and apparent use efficiency approach (*AUE*). The *ZnRec*_sum_ values are corrected for the uptake of Zn derived from the seed. For each plant, the values of *ZnRec*_sum_ and *AUE* were averaged (x-axis) and plotted against their difference (y-axis). The dotted line shows the bias and the dashed lines represent the 95% confidence interval (CI) of the Bland–Altman analysis.


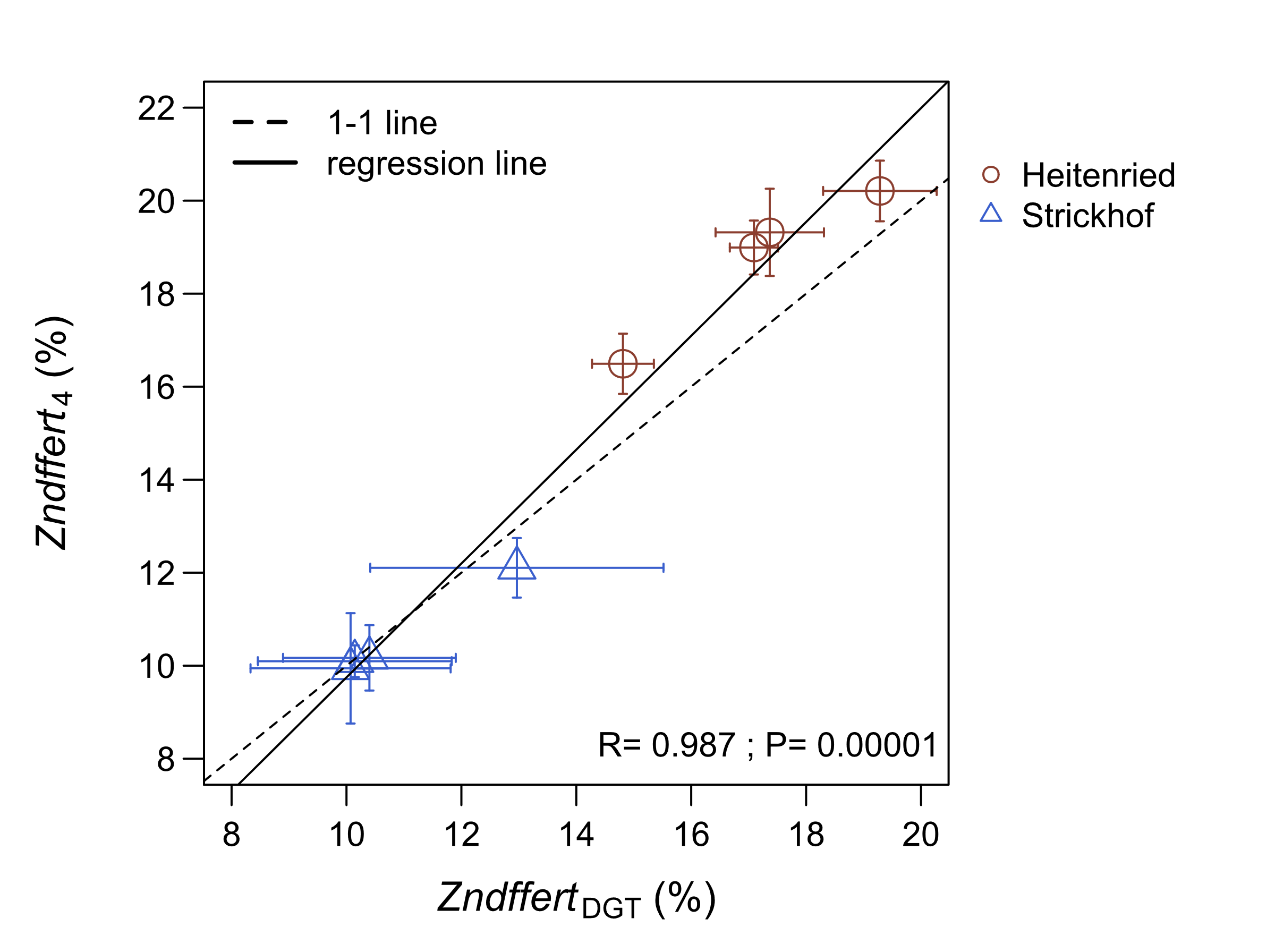


**Supplementary Figure S3.** Correlation between the proportion of DGT-extracted Zn that derived from the fertilizers (*Zndffert*_DGT_) on Heitenried soil and Strickhof soil incubated for 48 days after the addition of distinct Zn-containing fertilizers and proportion of Zn derived from the Zn-containing fertilizers in the ryegrass shoot at cut 4 (*Zndffert*_4_) subjected to the same treatment cultivated in the same soil in the same growth chamber. Values represent the mean values of *n* = 4 replicates ± standard deviation.

**Supplementary Table S1.** Dry weight (DW), Zn concentration, and Zn uptake in the Italian ryegrass shoots cultivated in a growth chamber and grown in Heitenried soil (pH = 4.9) and Strickhof soil (pH = 7.7) amended with distinct Zn- containing fertilizers measured at different cuts. Values represent the mean values of *n* = 4 replicates ± standard deviation

| **Soil** | **Treatment** | **Shoot dry weight** | | | |  | **Zn concentration in shoots** | | | |  | **Zn uptake in shoots** | | | |
| --- | --- | --- | --- | --- | --- | --- | --- | --- | --- | --- | --- | --- | --- | --- | --- |
|  |  | Cut 1 | Cut 2 | Cut 3 | Cut 4 |  | Cut 1 | Cut 2 | Cut 3 | Cut 4 |  | Cut 1 | Cut 2 | Cut 3 | Cut 4 |
|  |  | g DW kg^-1^ soil  μg kg^-1^ soil | | | |  | μg Zn g^-1^ DW shoots | | | |  | μg Zn kg^-1^ soil | | | |
|  |  |  | | | |  |  |  | | |  |  | | | |
| *Heitenried* | No Zn | 3.89 ± 1.40a | 2.96 ± 0.45a | 2.74 ± 0.37a | 3.52 ± 0.36ab |  | 56.3 ± 3.9a | 53.1 ± 1.3a | 59.8 ± 4.2a | 50.5 ± 3.4a |  | 222 ± 92a | 157 ± 21a | 162 ± 10a | 178 ± 25ab |
|  | ZnSO_4_ | 4.99 ± 0.59ab | 3.44 ± 0.45a | 2.77 ± 0.25a | 3.36 ± 0.33ab |  | 54.6 ± 1.9a | 57.9 ± 1.9a | 59.5 ± 4.0a | 53.5 ± 2.0a |  | 274 ± 41ab | 198 ± 20ab | 165 ± 18a | 180 ± 20ab |
|  | Sewage sludge | 5.56 ± 0.48b | 3.51 ± 0.35a | 2.75 ± 0.39a | 3.27 ± 0.21ab |  | 56.5 ± 2.7a | 59.2 ± 1.3a | 36.4 ± 3.4a | 52.3 ± 1.8a |  | 314 ± 35ab | 203 ± 20ab | 154 ± 14a | 171 ± 17ab |
|  | Poultry manure | 6.18 ± 0.32b | 3.69 ± 0.21a | 2.88 ± 0.37a | 3.14 ± 0.22a |  | 54.1 ± 1.1a | 56.5 ± 3.2a | 58.0 ± 4.1a | 49.6 ± 2.3a |  | 335 ± 13b | 208 ± 8b | 167 ± 20a | 155 ± 7a |
|  | Cattle manure | 4.55 ± 0.41ab | 3.66 ± 0.15a | 2.90 ± 0.15a | 3.84 ± 0.11b |  | 55.6 ± 1.3a | 59.0 ± 4.3a | 62.4 ± 2.5a | 52.3 ± 2.1a |  | 254 ± 27ab | 216 ± 25b | 181 ± 7a | 201 ± 11b |
|  |  |  |  |  |  |  |  |  |  |  |  |  |  |  |  |
| *Strickhof* | No Zn | 5.92 ± 0.65ab | 4.06 ± 0.16a | 3.02 ± 0.15ab | 3.32 ± 0.32a |  | 37.2 ± 2.8a | 40.6 ± 2.6a | 42.6 ± 2.2a | 32.6 ± 1.8a |  | 219 ± 20a | 165 ± 11ab | 129 ± 12ab | 108 ± 9ab |
|  | ZnSO_4_ | 6.30 ± 0.33ac | 3.88 ± 0.29a | 2.82 ± 0.20ab | 3.25 ± 0.30a |  | 40.9 ± 1.6ac | 43.8 ± 1.7a | 43.0 ± 1.8ab | 35.0 ± 1.3ab |  | 258 ± 16bc | 174 ± 15ab | 121 ± 12a | 114 ± 7ab |
|  | Sewage sludge | 6.92 ± 0.30c | 3.72 ± 0.40a | 2.96 ± 0.17ab | 3.00 ± 0.29a |  | 38.4 ± 1.5ab | 43.2 ± 1.1a | 43.6 ± 2.2ab | 33.6 ± 3.0a |  | 266 ± 16bc | 161 ± 19a | 129 ± 12ab | 100 ± 3a |
|  | Poultry manure | 6.84 ± 0.13bc | 4.06 ± 0.17a | 2.71 ± 0.09a | 2.72 ± 0.23a |  | 41.8 ± 1.2bc | 44.6 ± 3.8ab | 45.8 ± 2.9ab | 36.2 ± 2.3ab |  | 286 ± 5c | 181 ± 19ab | 124 ± 9a | 99 ± 14a |
|  | Cattle manure | 5.52 ± 0.52a | 3.94 ± 0.40a | 3.19 ± 0.23b | 3.08 ± 0.22a |  | 45.1 ± 2.6c | 51.9 ± 5.4b | 48.0 ± 2.7b | 39.4 ± 1.4b |  | 248 ± 15ab | 203 ± 21b | 153 ± 19b | 121 ± 6b |

For each soil, different letters in the same column indicate significant differences (*P* <0.05) among treatments.

**Supplementary Table S2**. Measured relative zinc isotope abundance (% of total Zn) in the soils extracted by plants and in the Zn-containing fertilizers used in this study. Values represent the mean values of *n* = 4 replicates ± standard deviation

| **Sources of Zn** |  | **^64^Zn** | **^66^Zn** | **^67^Zn** | **^68^Zn** | **^70^Zn** |
| --- | --- | --- | --- | --- | --- | --- |
| **Soil (%, estimated by plant extraction^a^)** | Heitenried | 46.76 ± 0.02 | 26.64 ± 0.05 | 7.98 ± 0.09 | 18.02 ± 0.05 | 0.60 ± 0.005 |
|  | Strickhof | 48.05 ± 0.08 | 27.25 ± 0.05 | 5.76 ± 0.01 | 18.33 ± 0.06 | 0.61 ± 0.006 |
|  |  |  |  |  |  |  |
| **Zn-containing fertilizer (%)^b^** | ZnSO_4_ | 48.85 ± 0.08 | 27.81 ± 0.07 | 4.06 ± 0.02 | 18.65 ± 0.04 | 0.62 ± 0.004 |
|  | Sewage sludge | 48.83 ± 0.02 | 27.80 ± 0.03 | 4.07 ± 0.02 | 18.67 ± 0.03 | 0.62 ± 0.002 |
|  | Poultry manure | 48.92 ± 0.03 | 27.79 ± 0.03 | 4.06 ± 0.02 | 18.61 ± 0.03 | 0.62 ± 0.003 |
|  | Cattle manure | 48.89 ± 0.03 | 27.81 ± 0.02 | 4.06 ± 0.01 | 18.62 ± 0.03 | 0.62 ± 0.006 |
|  |  |  |  |  |  |  |
| **Seed (%)^c^** |  | 49.17 | 27.73 | 4.04 | 18.45 | 0.61 |

^a^ Measured in HNO_3_ microwave digested Italian ryegrass shoots of the no Zn treatment at cut 4

^b^ Measured in HNO_3_ microwave digested fertilizers^[[1]](#footnote-1)^

^c^ According to the International Union of Pure and Applied Chemistry (IUPAC) Technical Report^[[2]](#footnote-2)^

**Supplementary Table S3.** Proportion of Zn derived from the seed (*Zndfseed*), Zn fertilizers (*Zndffert*) and soils (*Zndfsoil*) in the Italian ryegrass shoots cultivated in a growth chamber and grown in Heitenried soil (pH = 4.9) and Strickhof soil (pH = 7.7) amended with distinct Zn-containing fertilizers measured at different cuts. Values represent the mean values of *n* = 4 replicates ± standard deviation.

| **Soil** | **Treatment** | ***Zndfseed*** | |  | ***Zndffert*** | | | |  | ***Zndfsoil*** | | | |
| --- | --- | --- | --- | --- | --- | --- | --- | --- | --- | --- | --- | --- | --- |
|  |  | Cut 1 | Cut 2-4 |  | Cut 1 | Cut 2 | Cut 3 | Cut 4 |  | Cut 1 | Cut 2 | Cut 3 | Cut 4 |
|  |  |  | |  | % | | | |  | % | | | |
|  |  |  | |  |  |  | | |  |  | | | |
| *Heitenried* | No Zn | 5.62 ± 2.82a | 0 |  | 0 | 0 | 0 | 0 |  | 94.4 ± 2.8c | 100c | 100c | 100c |
|  | ZnSO_4_ | 5.27 ± 0.77a | 0 |  | 17.4 ± 1.3a | 17.1 ± 0.6a | 16.6 ± 0.5a | 16.5 ± 0.6a |  | 77.3 ± 0.6b | 82.9 ± 0.6b | 83.4 ± 0.5b | 83.5 ± 0.6b |
|  | Sewage sludge | 4.55 ± 0.50a  50aa | 0 |  | 20.0 ± 0.5b | 20.5 ± 0.4b | 19.1 ± 0.4b | 19.3 ± 0.9b |  | 75.4 ± 0.5ab | 79.5 ± 0.4a | 80.9 ± 0.4a | 80.7 ± 0.9a |
|  | Poultry manure | 4.24 ± 0.17a | 0 |  | 21.4 ± 0.3b | 20.6 ± 0.8b | 19.1 ± 0.7b | 20.2 ± 0.7b |  | 74.3 ± 0.5ab | 79.4 ± 0.8a | 80.9 ± 0.7a | 79.8 ± 0.7a |
|  | Cattle manure | 5.64 ± 0.68a | 0 |  | 20.3 ± 1.4b | 20.4 ± 0.8b | 19.5 ± 0.6b | 19.0 ± 0.6b |  | 74.0 ± 0.8a | 79.6 ± 0.8a | 80.5 ± 0.6a | 81.0 ± 0.6a |
|  |  |  | |  |  |  |  |  |  |  |  |  |  |
| *Strickhof* | No Zn | 6.38 ± 2.42a | 0 |  | 0 | 0 | 0 | 0 |  | 93.6 ± 2.4b | 100b | 100b | 100c |
|  | ZnSO_4_ | 5.43 ± 0.35a | 0 |  | 13.4 ± 2.3a | 12.1 ± 0.7a | 10.7 ± 1.2a | 12.1 ±0.6b |  | 81.1 ± 2.0a | 87.9 ± 0.7a | 89.3 ± 1.2a | 87.9 ± 0.6a |
|  | Sewage sludge | 5.26 ± 0.31a | 0 |  | 12.3 ± 1.0a | 9.9 ± 1.0a | 10.4 ± 1.8a | 9.9 ± 1.2a |  | 82.4 ± 1.0a | 90.1 ± 1.0a | 89.6 ± 1.8a | 90.1 ± 1.2b |
|  | Poultry manure | 4.88 ± 0.08a | 0 |  | 13.8 ± 3.5a | 9.6 ± 2.1a | 9.6 ± 1.6a | 10.1 ± 0.3a |  | 81.4 ± 3.5a | 90.4 ± 2.1a | 90.4 ± 1.6a | 89.9 ± 0.3b |
|  | Cattle manure | 5.64 ± 0.33a | 0 |  | 12.1 ± 0.3a | 10.5 ± 1.6a | 10.2 ± 1.9a | 10.2 ± 0.7a |  | 82.2 ± 0.3a | 89.5 ± 1.6a | 89.8 ± 1.9a | 89.8 ± 0.7b |

For each soil, different letters in the same column indicate significant differences (*P* <0.05) among treatments.

**Supplementary Table S4.** Zn uptake derived from the seeds (*QZndfseed*), Zn fertilizers (*QZndffert*), and from soils (*QZndfsoil*) in the Italian ryegrass shoots cultivated in a growth chamber and grown in Heitenried soil (pH = 4.9) and Strickhof soil (pH = 7.7) amended with distinct Zn- containing fertilizers measured at different cuts. Values represent the mean values of *n* = 4 replicates ± standard deviation. *QZndffert* values are corrected for the uptake of Zn derived from the seed

| **Soil** | **Treatment** | ***QZndfseed*** | | | |  | ***QZndffert*** | | | |  | ***QZndfsoil*** | | | |
| --- | --- | --- | --- | --- | --- | --- | --- | --- | --- | --- | --- | --- | --- | --- | --- |
|  |  | Cut 1 | Cut 2 | Cut 3 | Cut 4 |  | Cut 1 | Cut 2 | Cut 3 | Cut 4 |  | Cut 1 | Cut 2 | Cut 3 | Cut 4 |
|  |  | μg Zn kg^-1^ soil | | | |  | μg Zn g^-1^ DW shoots | | | |  | μg Zn kg^-1^ soil | | | |
|  |  |  | | | |  |  |  | | |  |  | | | |
| *Heitenried* | No Zn | 14.2 ± 12.1 | 0 | 0 | 0 |  | 0 | 0 | 0 | 0 |  | 208 ± 80a | 157 ± 21a | 162 ± 10b | 178 ± 25c |
|  | ZnSO_4_ | 14.2 | 0 | 0 | 0 |  | 48.0 ± 10.9a | 34.0 ± 4.5a | 27.2 ± 2.7a | 29.5 ± 2.2a |  | 211 ± 30a | 164 ± 16a | 137 ± 15ab | 150 ± 18ac |
|  | Sewage sludge | 14.2 | 0 | 0 | 0 |  | 63.1 ± 8.2ab | 42.0 ± 4.9ab | 29.4 ± 3.1ab | 32.5 ± 3.4ab |  | 237 ± 27a | 161 ± 15a | 125 ± 11a | 136 ± 16ab |
|  | Poultry manure | 14.2 | 0 | 0 | 0 |  | 71.3 ± 2.4b | 42.8 ± 2.0ab | 31.9 ± 3.7ab | 31.4 ± 1.5a |  | 247 ± 13a | 165 ± 7a | 135 ± 17a | 124 ± 6a |
|  | Cattle manure | 14.2 | 0 | 0 | 0 |  | 51.8 ± 8.4a | 44.1 ± 5.7b | 35.3 ± 1.2b | 38.1 ± 3.3b |  | 188 ± 19a | 172 ± 19a | 145 ± 6ab | 162 ± 8bc |
|  |  |  |  |  |  |  |  |  |  |  |  |  |  |  |  |
| *Strickhof* | No Zn | 14.0 ± 5.3 | 0 | 0 | 0 |  | 0 | 0 | 0 | 0 |  | 205 ± 20a | 165 ± 11a | 129 ± 12ab | 108 ± 9b |
|  | ZnSO_4_ | 14.0 | 0 | 0 | 0 |  | 34.9 ± 8.1a | 20.8 ± 0.9ab | 12.9 ± 2.0a | 13.8 ± 1.4b |  | 209 ± 8.3a | 153 ± 14a | 108 ± 10a | 100 ± 6ab |
|  | Sewage sludge | 14.0 | 0 | 0 | 0 |  | 32.7 ± 3.6a | 15.9 ± 2.6a | 13.5 ± 3.3a | 9.9 ± 1.1a |  | 219 ± 14a | 145 ± 17a | 116 ± 9.5ab | 90 ± 4a |
|  | Poultry manure | 14.0 | 0 | 0 | 0 |  | 39.5 ± 10.7a | 17.2 ± 2.7ab | 11.9 ± 2.0a | 10.0 ± 1.3a |  | 233 ± 8a | 164 ± 21a | 112 ± 8.7ab | 89 ± 12a |
|  | Cattle manure | 14.0 | 0 | 0 | 0 |  | 30.1 ± 2.3a | 21.1 ± 2.6b | 15.4 ± 1.4a | 12.3 ± 1.0ab |  | 204 ± 12a | 182 ± 21a | 138 ± 20b | 109 ± 6b |

For each soil, different letters in the same column indicate significant differences (*P* <0.05) among treatments.

**Supplementary Table S5.** Recovery of Zn derived from the studied fertilizers in the Italian ryegrass shoot (sum of 4 cuts) cultivated in a growth chamber and grown in Heitenried soil (pH = 4.9) and Strickhof soil (pH = 7.7) with distinct Zn-containing fertilizers (*ZnRec*_sum_). Values represent the mean values of *n* = 4 replicates ± standard deviation. All values are corrected for the uptake of Zn derived from the seed

| **Soil** | **Treatment** | ***ZnRec*_sum_** | | | |
| --- | --- | --- | --- | --- | --- |
|  |  | Cut 1 | Cut 2 | Cut 3 | Cut 4 |
|  |  | % of Zn input | | | |
|  |  |  | | | |
| *Heitenried* | ZnSO_4_ | 3.38 ± 0.77a | 2.39 ± 0.32a | 1.92 ± 0.19a | 2.08 ± 0.16a |
|  | Sewage sludge | 4.15 ± 0.54ab | 2.76 ± 0.32a | 1.94 ± 0.21a | 2.14 ± 0.22a |
|  | Poultry manure | 4.72 ± 0.16b | 2.84 ± 0.14a | 2.11 ± 0.25a | 2.08 ± 0.10a |
|  | Cattle manure | 3.32 ± 0.54a | 2.83 ± 0.36a | 2.26 ± 0.08a | 2.44 ± 0.21a |
|  |  |  |  |  |  |
| *Strickhof* | ZnSO_4_ | 2.46 ± 0.57a | 1.47 ± 0.07b | 0.91 ± 0.14a | 0.97 ± 0.10b |
|  | Sewage sludge | 2.15 ± 0.24a | 1.05 ± 0.17a | 0.89 ± 0.22a | 0.65 ± 0.07a |
|  | Poultry manure | 2.61 ± 0.71a | 1.14 ± 0.18ab | 0.79 ± 0.13a | 0.66 ± 0.09a |
|  | Cattle manure | 1.93 ± 0.15a | 1.35 ± 0.17ab | 0.99 ± 0.09a | 0.79 ± 0.06a |

*ZnRec*_sum_ was calculated as the uptake of Zn derived from the studied fertilizer in ryegrass shoots at cuts 1-4 divided by the total input of Zn with the fertilizer.

For each soil, different letters in the same column indicate significant differences (*P* <0.05) among treatments.

1. Dürr-Auster, T.; Wiggenhauser, M.; Zeder, C.; Schulin, R.; Weiss, D. J.; Frossard, E., The use of Q-ICPMS to apply enriched zinc stable isotope source tracing for organic fertilizers. *Front. Plant Sci.* **2019**, 10, 1382. [↑](#footnote-ref-1)
2. Meija, J.; Coplen, T. B.; Berglund, M.; Brand, W. A.; De Bièvre, P.; Gröning, M.; Holden, N. E.; Irrgeher, J.; Loss, R. D.; Walczyk, T., Atomic weights of the elements 2013 (IUPAC Technical Report). *Pure Appl. Chem.* **2016,** *88* (3), 265-291. [↑](#footnote-ref-2)
